# Supplementary material for: Phenotypic quantification of root spatial distribution along circumferential direction for field paddy-wheat
Source: PLoS One. 2023 Jul 7;18(7):e0279353. doi: 10.1371/journal.pone.0279353 (PMC10328375; doi:10.1371/journal.pone.0279353)
Supplement: Attachment — Submitted filename: comments_PlosOne.docx [file pone.0279353.s003.docx]

The manuscript titled “**Phenotyping for circumferential expansion of field paddy-wheat root systemarchitecture**” emphasize on wheat root system with reference to circumferential orientations. Using three fertilization rates and experiment was designed to quantify dynamic root system architecture (RSA). With field root *in situ* samplings and RSA digitization, authors findings are presented in this manuscript.

Introduction and material method section is written well.

In result section table-2, last column is not understandable, either it is p-value or t-value. If it is p-value, that is non-significant and if it is –value that is also non-significant. Without clarification of this aspect, this table is not able to be published.

Variation has not been well explained in reference to p-value, confidence level etc; so under these condition, such results are unacceptable. Author should justify the results with p-value using analysis of variance models.

In line # 243, author mentioned one-factorial anova, but I could not see that in table with p-value.

The overall conclusion of the study is missing and describe the novelty of your research .

Under such conditions, this study is not publishable unless the above mentioned suggestions are addressed properly.
