# Supplementary material for: Phenotypic quantification of root spatial distribution along circumferential direction for field paddy-wheat
Source: PLoS One. 2023 Jul 7;18(7):e0279353. doi: 10.1371/journal.pone.0279353 (PMC10328375; doi:10.1371/journal.pone.0279353)
Supplement: Attachment — Submitted filename: Response to Reviewers.docx [file pone.0279353.s004.docx]

Dear editor,

Thank you very much for providing me the opportunity to revise the manuscript.

We have revised the above-mentioned manuscript carefully according to the comments from the Journal and reviewers. The responses to the Journal’ requirements and reviewer’s comments and changes made are listed as follows:

**Journal’s Requirement 1:**

Please ensure that your manuscript meets PLOS ONE's style requirements, including those for file naming. The PLOS ONE style templates can be found at

https://journals.plos.org/plosone/s/file?id=wjVg/PLOSOne_formatting_sample_main_body.pdf and

<https://journals.plos.org/plosone/s/file?id=ba62/PLOSOne_formatting_sample_title_authors_affiliations.pdf>

**Responses and Changes Made:**

According to the PLOS ONE's style requirements, we have changed the article structure consist of abstract, introduction, materials and methods, results, discussion into abstract, introduction, materials and methods, results and discussion, conclusion. And the title_authors_affiliations were also modified in title page as required.

**Journal’s Requirement 2:**

In your Data Availability statement, you have not specified where the minimal data set underlying the results described in your manuscript can be found. PLOS defines a study's minimal data set as the underlying data used to reach the conclusions drawn in the manuscript and any additional data required to replicate the reported study findings in their entirety. All PLOS journals require that the minimal data set be made fully available. For more information about our data policy, please see <http://journals.plos.org/plosone/s/data-availability.>

Upon re-submitting your revised manuscript, please upload your study’s minimal underlying data set as either Supporting Information files or to a stable, public repository and include the relevant URLs, DOIs, or accession numbers within your revised cover letter. For a list of acceptable repositories, please see http://journals.plos.org/plosone/s/data-availability#loc-recommended-repositories. Any potentially identifying patient information must be fully anonymized.

**Responses and Changes Made:**

According to the PLOS ONE's data requirements, we rewritten it in the part of the materials and methods marked with blue font (Line 135-138), also we rearranged all the relevant data and put them in the Supporting Information files (S1 Data).

**Reviewer #1**

**Comment of reviewer 1:**

The manuscript has been wisely put together with very clear vision and results. The use of figures is very intelligent. Overall it reflects a strong scientific research article.
The manuscript has a clear description of results with extensively defined figures.

**Responses and Changes Made:**

Thanks very much for the comments of the reviewer. We will continue to work hard to present our future studies in a scientific, understandable and wise way. We hope readers in this field or other fields all can get some useful information from it.

**Reviewer #2**

**Comment 1 of Reviewer 2:**

overall the manuscript is well written but major revision requires. it will be accepted after revision. NO novelty statement was nor provided and some statistical error in your manuscript. result and material method was written well.

The manuscript titled “**Phenotyping for circumferential expansion of field paddy-wheat root systemarchitecture”** emphasize on wheat root system with reference to circumferential orientations. Using three fertilization rates and experiment was designed to quantify dynamic root system architecture (RSA). With field root in situ samplings and RSA digitization, authors findings are presented in this manuscript.

Introduction and material method section is written well.

In result section table-2, last column is not understandable, either it is p-value or t-value. If it is p-value, that is non-significant and if it is p-value that is also non-significant. Without clarification of this aspect, this table is not able to be published.

**Responses and Changes Made:**

According to the reviewer’s comments, we revised Table 2 marked in blue font and listed the average value and coefficient of variation of each indicator as the comparison object, then we constructed the relationship function between different nitrogen application rates. The P value was not listed, but where there were significant differences, the asterisk (p<0.05) or double asterisk (p<0.01) was used to mark the corresponding R² value, and we put the data in the Supporting Information files (S2 Data).

**Comment 2 of Reviewer 2**

Variation has not been well explained in reference to p-value, confidence level etc; so under these condition, such results are unacceptable. Author should justify the results with p-value using analysis of variance models.

**Responses and Changes Made:**

According to the reviewer’s comments, we analyzed the novel indicators with p-value using One-factorial ANOVA and justified the results in the part of Results and discussions marked with blue font (Line363-370 ).

**Comment 3 of Reviewer 2**

In line # 243, author mentioned one-factorial anova, but I could not see that in table with p-value.

**Responses and Changes Made:**

According to the reviewer’s comments, we illustrated p<0.05 and p<0.01 with an asterisk and double asterisk, respectively ( Table 1 and Table 2).

**Comment 4 of Reviewer 2**

The overall conclusion of the study is missing and describe the novelty of your research .Under such conditions, this study is not publishable unless the above mentioned suggestions are addressed properly.

**Responses and Changes Made:**

According to the reviewer’s comments, we supplemented the conclusion in which we described the novelty of our research in the part of the Conclusions marked with blue font (Line 376-393).

**Reviewer #3**

**Comment 1 of Reviewer 3:**

The present manuscript entitled as “Phenotyping for circumferential expansion of field paddy-wheat root system architecture” describes the findings of a field experiment on wheat under three fertilization rates and provided some new parameters to quantify the dynamics of the root system architecture along its orientations divided into eight parts with visualization technology that consists of root in situ samplings. Authors collected plant samples during growing season 2019-20. The research idea and objectives are unique and research methodology is well organized according to the objectives. However, there are some flaws and drawbacks in the present version of article.
The manuscript is poorly written, there are some sentences which are not meaningful or don’t make any clear sense for the readers. So, I recommend that the current version of the submitted manuscript should be very carefully revised and upgraded. After critical and major revision in the writings, I recommend to accept this manuscript. I also, have some other major concerns which are appended below:
**Responses and Changes Made:**

According to the reviewer’s comments, the manuscript has been revised upgraded carefully, especially for writings. We also discussed it with a foreign assistant professor to reduce its writing problems and improve the readability. We did not to mark the above changes with colored fonts, because the whole article has been modified. For other concerns, we have made corresponding modifications and marked with blue font.

**Comment 2 of Reviewer 3:**

Line 4: you used the word “hidden half” in abstract, does it indicates roots? If so then it’s better to use roots to make the sentence easy to understand.
**Responses and Changes Made:**

According to the reviewer’s comments, we have revised "hidden half" with roots in the abstract for the word “hidden half” in abstract indicates roots, and the modified part was marked with blue font (Line 4).

**Comment 3 of Reviewer 3:**

Line 11: It is not clear whether you conducted research on paddy and wheat or on wheat alone that was grown in paddy soil, make it clear. You didn’t mention Nitrogen fertilizer rate used in you treatments?

**Responses and Changes Made:**

According to the reviewer’s comments, to clarify this problem, we illustrated it in the parts of the Materials and methods (Line 100-103) that we conducted the research on wheat alone grown in paddy soil. For the Nitrogen fertilizer rate used in our treatments, we also revised it in the parts of the Materials and methods (Line 108-111). And the modified part was marked with blue font.

**Comment 4 of Reviewer 3:**

Lines 11-12: You mentioned in manuscript that “we provided five new to quantify the dynamics of the root system architecture”; what did you provide new?

**Responses and Changes Made:**

According to the reviewer’s comments, we concluded it in the part of the Results and Discussions (Line 327-335) and Conclusions (Line376-388) marked with blue font. In short, the new indicators come from a new analysis angle combined with previous root research methods and quantitative indicators.

**Comment 5 of Reviewer 3:**

Line 20: you didn’t mention nitrogen rate and you stated about N70 and N130 nitrogen treatment in your results, provide treatment details clearly.

**Responses and Changes Made:**

According to the reviewer’s comments, we supplied the treatments of nitrogen fertilizer rates in detail in the parts of the Materials and methods marked with blue font (Line 108-111), shown as “Before sowing, the application of diammonium phosphate was 375 kg∙hm2, potassium chloride was 375 kg∙hm2, and the applications of urea were 70 kg∙hm2, 100 kg∙hm2 and 130 kg∙hm2, which represented the three N application rates and were named N70, N100, and N130 respectively.”

**Comment 6 of Reviewer 3:**

Line 49-51: clearly state about “angle”.

**Responses and Changes Made:**

According to the reviewer’s comments, we changed the angles into the root growth angles (Line 49-51) stated clearly in Line 38-41, which marked with blue font. The angle used in the manuscript implies the root growth angle between the root growth direction and the horizontal plane.

**Comment 7 of Reviewer 3:**

Line 63: what do you mean by “Depth50 and Width50”?

**Responses and Changes Made:**

According to the reviewer’s comments, we revised this problem and deleted it because it indicates a lack of use. The Depth50 and Width50 represent the vertical and horizontal centroids of root distribution, respectively [Teramoto S and Uga Y, 2020].

**Comment 8 of Reviewer 3:**

Line 89-90: Provide country name as well. Replace light with sunlight. Provide groundwater level if possible.

**Responses and Changes Made:**

According to the reviewer’s comments, we provided the country name and replaced light with sunlight marked with blue font (Line 89-90). However, we did not find the groundwater level of this area in the authoritative data, so we did not supplement the data on the groundwater level.

**Comment 9 of Reviewer 3:**

Line 93-94: Make this sentence grammatically correct.

**Responses and Changes Made:**

According to the reviewer’s comments, we changed this sentence into “In the field, the rice-wheat rotation period is long, and a relatively hard plow bottom layer has formed at a depth of 10-12 cm soil layer due to alternating floods and droughts and mechanized operation.” Marked with blue font (Line 92-95).

**Comment 10 of Reviewer 3:**

Line 134-135: You didn’t mention three nitrogen rate in methodology, provide clear details of your treatments?

**Responses and Changes Made:**

According to the reviewer’s comments, we provided the three nitrogen fertilizer rate in detail in the parts of the Materials and methods marked with blue font (Line 108-111).

**Comment 11 of Reviewer 3:**
Line 179: Check and correct spellings

**Responses and Changes Made:**

According to the reviewer’s comments, we checked and corrected the “Convsex ” with “Convex” marked with blue font (Line 181).

**Comment 12 of Reviewer 3:**
Line 201: Check and correct the formula.

**Responses and Changes Made:**

According to the reviewer’s comments, we checked and corrected the “* ” with “x” in the formula (Line 203).

**Comment 13 of Reviewer 3:**
Line 234: Use superscript for mm and do same for all manuscript.

**Responses and Changes Made:**

According to the reviewer’s comments, we used superscript for mm and checked and did the same for all the manuscript.

**Comment 14 of Reviewer 3:**
Line 255-256: Provide space between words and parenthesis, do same for all manuscript.

**Responses and Changes Made:**

According to the reviewer’s comments, we provided space between words and parenthesis, and checked and did the same for all the manuscript.

**Comment 15 of Reviewer 3:**
Line 351: What new indicators did you observe, write those.

**Responses and Changes Made:**

According to the reviewer’s comments, we introduced them in the part of the Materials and methods (Line 166-204) and concluded them in the part of the Abstract (Line 11-22) and the Conclusions (Line 376-393). Changes made above were marked with blue font. We provided five new indicators (PRVP, CHARVP, WDRRSP, PRHP and RLSS) to quantify the dynamics of the root system architecture (RSA) along its eight-part circumferential orientations with visualization technology. And the visualization technology consists of in-situ field root samplings, RSA digitization, and reconstruction according to previous research. In short, the new indicators come from a new analysis angle combined with previous root research methods and quantitative indicators.
