# Supplementary material for: Phenotypic quantification of root spatial distribution along circumferential direction for field paddy-wheat
Source: PLoS One. 2023 Jul 7;18(7):e0279353. doi: 10.1371/journal.pone.0279353 (PMC10328375; doi:10.1371/journal.pone.0279353)
Supplement: Attachment — Submitted filename: Response to Reviewers.DOCX [file pone.0279353.s005.DOCX]

Dear editor,

Thank you very much for providing me the opportunity to revise the manuscript again.

We have revised the above-mentioned manuscript carefully according to the comments from the Journal and reviewers. The responses to the Journal’ requirements and reviewer’s comments and changes made are listed as follows:

**Journal Requirements:**

Please review your reference list to ensure that it is complete and correct. If you have cited papers that have been retracted, please include the rationale for doing so in the manuscript text, or remove these references and replace them with relevant current references. Any changes to the reference list should be mentioned in the rebuttal letter that accompanies your revised manuscript. If you need to cite a retracted article, indicate the article’s retracted status in the References list and also include a citation and full reference for the retraction notice.

**Responses and Changes Made:**

According to the Journal’s requirements, we reviewed our reference list and found that the reference No. 68 has gone on the journal’s website (Chen J, Wang P, Ma ZM, Lyu XD, Liu TT, Siddique KHM. Optimum water and nitrogen supply regulates root distribution and produces high grain yields in spring wheat (Tritium aestivum L.) under permanent raised bed tillage in arid northwest China. Soil Till Res. 2018; 181:117-126.). So we removed and replaced it with a relevant current reference, also modified the corresponding context in the manuscript marked with the blue font (L361-363, Line 580-583).

**Reviewer #2**

**Comment of reviewer 2:**

Appreciation for authors that they incorporate all the objection carefully. This is strong enough to meet the potential to be published. i strongly recommended the publication of this paper

**Responses and Changes Made:**

Thanks very much for the comments and valuable suggestions of the reviewer for improving this manuscript. We are all glad that our manuscript revision has satisfied the reviewer.

**Reviewer #4:**

**Comment 1 of Reviewer 4:**

I have gone through the whole manuscript very thoroughly and found that the author has written it very extensively and descriptively.

**Responses and Changes Made:**
We agree with the reviewer’s comments, and we hypothesize that the indicators describing the characteristics of root system architecture still need to be supplemented. Taking the root system of field paddy-wheat as the research object, we propose five new indicators characterizing the root spatial distribution characteristics along the circumferential directions based on the previous research methods. And the test results show that the newly proposed indicators can quantitatively describe the characteristics of spatial root distribution along the circumferential directions. However, as we mentioned in the conclusion, fewer samples, only one year of the experiment, and only one wheat variety are shortcomings in writing it very affirmatively and specifically.

**Comment 2 of Reviewer 4:**

The title should be reframed as it looks like a simple sentence. It should be catchy, attractive and reflective of the research hypothesis.

**Responses and Changes Made:**

According to the reviewer’s comments, we changed the title “Phenotyping for circumferential expansion of field paddy-wheat root system architecture” to “Phenotypic quantification of root spatial distribution along circumferential directions for field paddy-wheat” after carefully thinking and discussing.

**Comment 3 of Reviewer 4:**
The abstract was written very scantily. It should be required. Therefore, please rewrite the abstract with proper hypotheses, the origin of study with clear-cut objectives, a brief methodology, and finally, results with proper justification and clarity.

**Responses and Changes Made:**

According to the reviewer’s comments, we rewrote the abstract, supplied the hypotheses (Lines 7-8), and refined the goals and methods (Lines 11-16), all marked with blue font. We framed the abstract as background (Lines 2-6), hypotheses and illustrations (Lines 7-11), goals and methods (Lines 11-16), results (Lines 16-22), conclusions and prospects (Lines 22-25).

**Comment 4 of Reviewer 4:**

The amount of rainfall received during the experimental period was important in determining crop growth and development, and no irrigation was provided during the growing seasons. You have mentioned that plant roots are essential for water and nutrient absorption, anchoring, mechanical support, metabolite storage, and interaction with the surrounding soil environment. As a result, I believe that water budgeting is necessary to justify the hypothesis because water is an important factor that influences root growth and development in field conditions.

**Responses and Changes Made:**

Thanks for the reviewer’s comments; Nanjing locates in the middle and lower reaches of the Yangtze River, with an annual average rainfall of about 1000 mm and high groundwater levels. The growth of field wheat here mainly depends on rainfall without irrigation. And this study mainly proposes new quantitative indicators of wheat root growth distribution growing in natural conditions. The test data used belonged to the same growth cycle, so we did not consider the impact of rainfall on root growth during the experiment. Also, we did not record rainfall data or soil moisture content during sampling time. According to the reviewer’s comments, we searched the rainfall data from the government's website and list them from 2010-2019 in the below chart (<http://www.jiangsu.gov.cn/col/col76741/index.html>), for the rainfall data of 2020 was not published. And the data marked with red font covered the sampling time. We think it may not be essential to supply this part in the manuscript.

| Year | Jan | Feb | Mar | Apr | May | Jun | Jul | Aug | Sep | Oct | Nov | Dec | Total |
| --- | --- | --- | --- | --- | --- | --- | --- | --- | --- | --- | --- | --- | --- |
| 2010 | 18.8 | 115.6 | 117.8 | 197.9 | 56.1 | 62.1 | 343.3 | 142.1 | 181.1 | 32.1 | 7.6 | 24 | 1298.4 |
| 2011 | 10.8 | 17.2 | 43.2 | 11.6 | 40.6 | 312.9 | 278 | 284.3 | 12.6 | 28.7 | 21.3 | 15.8 | 1077 |
| 2012 | 21 | 73.3 | 79.3 | 56.2 | 62.4 | 17.8 | 176.4 | 198.3 | 68.7 | 54.6 | 42.8 | 66.4 | 917.2 |
| 2013 | 17.8 | 69.9 | 42.9 | 22.8 | 110.1 | 172.6 | 229.6 | 115.5 | 67 | 22.4 | 17.2 | 10.6 | 898.4 |
| 2014 | 20.6 | 121.2 | 68.4 | 97.6 | 26.3 | 111.8 | 263.5 | 158.8 | 89.2 | 32 | 97.9 | 3.8 | 1091.1 |
| 2015 | 29.9 | 58.1 | 104.8 | 121.6 | 96.1 | 661.5 | 258 | 187.4 | 63.6 | 61.6 | 110.7 | 12.3 | 1765.6 |
| 2016 | 62.8 | 31.1 | 40 | 155.4 | 119.6 | 186.2 | 477.3 | 78.7 | 187.4 | 308 | 95.4 | 65.8 | 1807.7 |
| 2017 | 59.3 | 38.8 | 55.1 | 117.9 | 83.9 | 309.1 | 99.6 | 217.1 | 176.4 | 81 | 7.4 | 9.5 | 1255.1 |
| 2018 | 106 | 44.9 | 120.7 | 61 | 142.8 | 49.1 | 182.3 | 273.6 | 66.2 | 37.5 | 65.6 | 117.4 | 1267.1 |
| 2019 | 51.5 | 90.4 | 37.6 | 51.5 | 41.1 | 109.2 | 63.9 | 123 | 51.4 | 1.4 | 41.7 | 59.1 | 721.8 |

**Comment 5 of Reviewer 4:**

Figure quality may be improved for more clarity.

**Responses and Changes Made:**

The figures uploaded are compressed according to the requirements of the journal. According to the reviewer’s comments, we uploaded clearer pictures in supporting information.

**Comment 6 of Reviewer 4:**

The conclusion is very condensed; there is scope to improve it briefly as per the salient achievement of the study. Hence, it can be elaborated with final recommendations.

**Responses and Changes Made:**

According to the reviewer’s comments, we rearranged the conclusion marked with blue font (Lines 377-386).
